# Ankle strategy assistance to improve gait stability using controllers based on in-shoe center of pressure in 2 degree-of-freedom powered ankle–foot orthoses: a clinical study

**DOI:** 10.1186/s12984-022-01092-6

**Published:** 2022-10-25

**Authors:** Ho Seon Choi, Yoon Su Baek, Hyunki In

**Affiliations:** 1 Center for Healthcare Robotics, Korea Institute of Science and Technology, Seoul, 03722 South Korea; 2 School of Mechanical Engineering, Yonsei University, Seoul, 02792 South Korea

**Keywords:** Powered ankle–foot orthosis, Ankle strategy, Gait stability, Eversion, Subtalar joint, Local dynamic stability, Kinematic variability, Center of pressure

## Abstract

**Background:**

Although the ankle strategy is important for achieving frontal plane stability during one-leg stance, previously developed powered ankle–foot orthoses (PAFOs) did not involve ankle strategies because of hardware limitations. Weakness of movement in frontal plane is a factor that deteriorates gait stability and increases fall risk so it should not be overlooked in rehabilitation. Therefore, we used PAFO with subtalar joint for frontal plane movement and tried to confirm that the existence of it is important in balancing through clinical experiments.

**Methods:**

We developed a proportional CoP controller to assist ankle strategy or stabilizing moment and enhance eversion to compensate for the tilting moment with 2 *dof* PAFO. It was true experimental study, and we recruited seven healthy subjects (30 ± 4 years) who did not experience any gait abnormality participated in walking experiments for evaluating the immediate effect of subtalar joint of PAFO on their gait stability. They walked on the treadmill with several cases of controllers for data acquisitions. Indices of gait stability and electromyography for muscle activity were measured and Wilcoxon signed-rank tests were used to identify meaningful changes.

**Results:**

We found that subjects were most stable during walking (in terms of largest Lyapunov exponents, p < 0.008) with the assistance of the PAFO when their electromyographic activity was the most reduced (p < 0.008), although postural sway increased when a proportional CoP controller was used to assist the ankle strategy (p < 0.008). Other indices of gait stability, kinematic variability, showed no difference between the powered and unpowered conditions (p > 0.008). The results of the correlation analysis indicate that the actuator of the PAFO enhanced eversion and preserved the location of the CoP in the medial direction so that gait stability was not negatively affected or improved.

**Conclusions:**

We verified that the developed 2 *dof* PAFO assists the ankle strategy by compensating for the tilting moment with proportional CoP controller and that wearer can walk in a stable state when the orthosis provides power for reducing muscle activity. This result is meaningful because an ankle strategy should be considered in the development of PAFOs for enhancing or even rehabilitating proprioception.

*Trial registration* 7001988-202003-HR-833-03

## Background

Rehabilitation is the process by which patients or elderly individuals who have experienced loss or impairment of their motor ability try to regain it through training. A major element that rehabilitation attempts to recover is muscle strength, but the restoration of proprioception, which provides sensory information on limb position, joint speed and acceleration, and posture perception, is also important [1]. Proprioception decreases with aging [2, 3] and may also deteriorate with injury or disease such as stroke [4, 5]. It is also involved in movement stability; when proprioception of the ankle joint decreases or worsens, postural sway increases, and gait stability deteriorates, which increases the risk of falling [6, 7].

Various rehabilitation systems are being developed to prevent fall, and powered ankle–foot orthosis (PAFO) is one of them. PAFOs are wearable robots that helps rotate the wearer’s ankle joint [8]. Usually, they have a talocrural joint as an only axis, which is responsible for rotation in the sagittal plane. And they assist in providing the propulsion required for a walker to move forward. The purpose of these 1 degree-of-freedom (*dof*) PAFOs are to aid in propulsion, and many studies are being conducted to optimize the controller and minimize the objective functions related to muscle activity or metabolic rate [9–12]. However, as the performance of rehabilitation devices improve enough to be applied in the clinic and they are becoming sufficiently safe, studies on PAFOs which are focused on improving the wearer’s gait stability rather than providing simple assistance is becoming more common [13–15]. The goal of these qualitative rehabilitations is to restore the walking ability of the patients in a way that minimizes the risk of falling, rather than focusing only on moving forward, when the wearer’s ability to walk is impaired due to an accident or disease.

To properly restore the function of the ankle joint, it is necessary to consider the configuration of the joint. The ankle has a subtalar joint that rotates in the frontal plane in addition to a talocrural joint and its function is closely related to stability, the restoration of which is a goal of rehabilitation [16]. The subtalar joint preserves gait stability by controlling the rotation of the center of mass (CoM) in the frontal plane, which is known as the ankle strategy or foot tilt strategy [17, 18]. It generates a stabilizing moment that compensates for the tilting moment caused by the misalignment of the projection of the CoM, which is located in the trunk, on the ground and the plantar center of pressure (CoP) through eversion so that an individual can walk in a stable state. Approximately 80% of the gait cycle, excluding initial and terminal double support, involves a one-leg stance [19], so the ankle strategy performed during this period is very important in reducing the risk of falls of walkers. In fact, the reason for the deterioration of gait stability with aging is the decrease in the range of motion of eversion along with the weakening of the plantar flexion [20]. And this shows that ankle movement in the frontal plane considerably contributes to the prevention of fall risk.

However, the PAFOs that have been developed thus far have focused just on the talocrural joint. Rehabilitation performed in the absence of the subtalar joint does not enable complete recovery of proprioception, and learning of ankle strategy is not easily achieved, so there is a possibility that the patient won’t return to the pre-injury state after rehabilitation. To solve this problem, we fabricated a PAFO with both talocrural and subtalar joints [21] and used two pneumatic artificial muscles (PAMs) to simultaneously assist plantar flexion during propulsion and eversion when creating a stabilizing moment. Additionally, through comparative experiments with a 1 *dof* PAFO, we proved that eversion has a positive effect on reducing postural sway during walking with PAFOs [22]. However, since we measured simple postural sway only, we could not analyze how the power provided by the PAMs affects gait stability of the wearers.

Gait stability cannot be fully evaluated by assessing outward fluctuations such as postural sway, and it is usually assessed by examining cycle variation or local dynamic stability. Cycle variation is quantified by calculating the kinematic variability between cycles during cyclical movement [23]. Local dynamic stability is a method used to evaluate stability that involves measuring how the magnitude of a deviation increases as a cyclical movement proceeds after an initial external perturbation to the system [24]. Both are actively used to evaluate gait stability and the performance of exoskeleton robots, and both are directly related to the risk of falling, which is caused by deterioration of stability [25, 26]. Although we have already confirmed that the developed 2 *dof* PAFO affects postural sway in positive ways with previous study [22], we wanted to know whether the subtalar joint, which is included in the 2 *dof* PAFO, actually assists the ankle strategy of the wearers and generates a stabilizing moment to compensate for the tilting moment and preserve gait stability which were evaluated with not only outward fluctuations with postural sway but also indices for gait stability like kinematic variability or local dynamic stability.

It was proved that the characteristics of assistance from PAFO affect the wearers’ stability and that it might be more important than proper foot placement [27–29]. Although these studies are about experiments or simulations conducted with prosthetic foot or 1 *dof* PAFO, but they were enough to show the importance of assistance characteristics in wearers’ gait stability. So, considering that the fall risk is related to the weakening of the evertor [20], it is also meaningful to examine the wearers’ gait stability according to the characteristics of the assistance provided by 2 *dof* PAFO with subtalar joint as the rotation axis. In fact, in order to preserve stability during steady state gait, it is necessary to correct errors that occurred during foot placement, which can be implemented by an active ankle strategy by shifting the CoP in the medial–lateral direction [30]. So, if the assistance of 2 *dof* PAFO is conducted by designing proper controller, it helps to shift the CoP in a direction that compensates the tilting moment by assisting ankle strategy, or to provide a stabilizing moment so that it is expected that wearer can perform a stable gait.

In this study, we examined how the power provided by the 2 *dof* PAFO affects the gait stability of wearers using indices such as local dynamic stability and kinematic variability. In general, the trajectory of the CoP in the global coordinates is said to be positioned in more laterally to improve gait stability. However, in the case of in-shoe CoP measured in the local coordinate system of the sole during one-leg stance, the trajectory tends to move in a more medial direction during eversion because of the decrease of tilting moment [31]. We found in a previous study that the power generated by the PAM of a 1 *dof* PAFO for assisting plantar flexion causes the in-shoe CoP to move in the lateral direction, resulting in an increase in the tilting moment and subsequent postural sway, and that the power of the PAM in a 2 *dof* PAFO that is used to strengthen eversion compensates for that phenomenon. We used phase-based controllers (PBC) for previous study which is normally used for PAFOs but in this paper with these findings we developed controllers for PAMs based on the lateral deviation of the in-shoe CoP trajectory caused by propulsion assistance. Through the clinical experiments, we wanted to prove that the PAMs of 2 *dof* PAFO mitigate the deterioration of stability when stabilizing moments are appropriately provided to the wearer based on this algorithm. These experiments aimed to understand whether the ergonomic properties of the 2 *dof* PAFO could sufficiently support the wearer’s ankle strategy in the aspect of the gait stability.

## Methods

### 2 degree-of-freedom powered ankle–foot orthosis

#### Description of orthosis

**Fig. 1:**
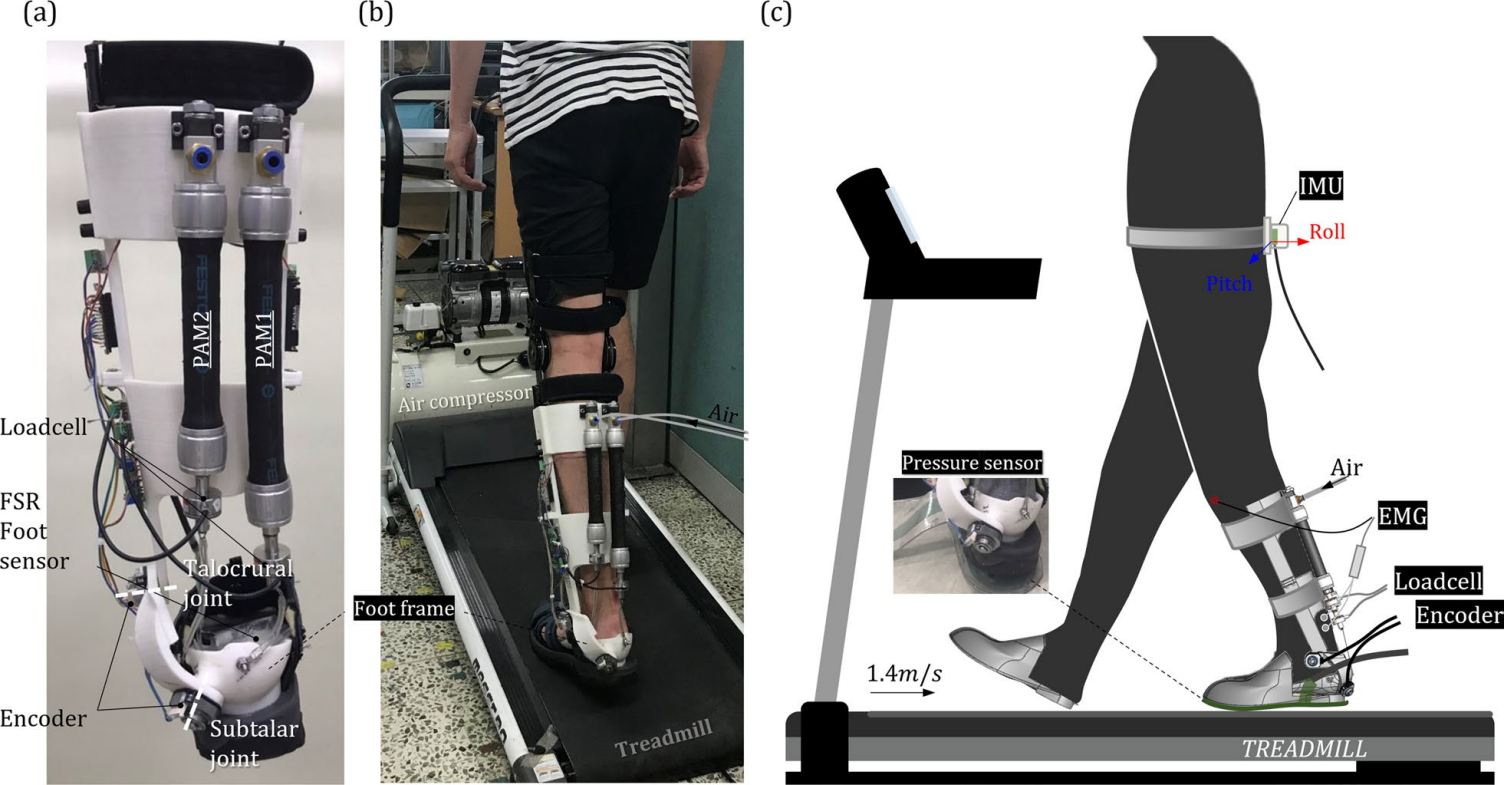
**a** Developed 2 dof PAFO with talocrural and subtalar joints. **b** Subject walking on the treadmill and wearing the 2 dof PAFO. **c** Experimental setup

**Fig. 2:**
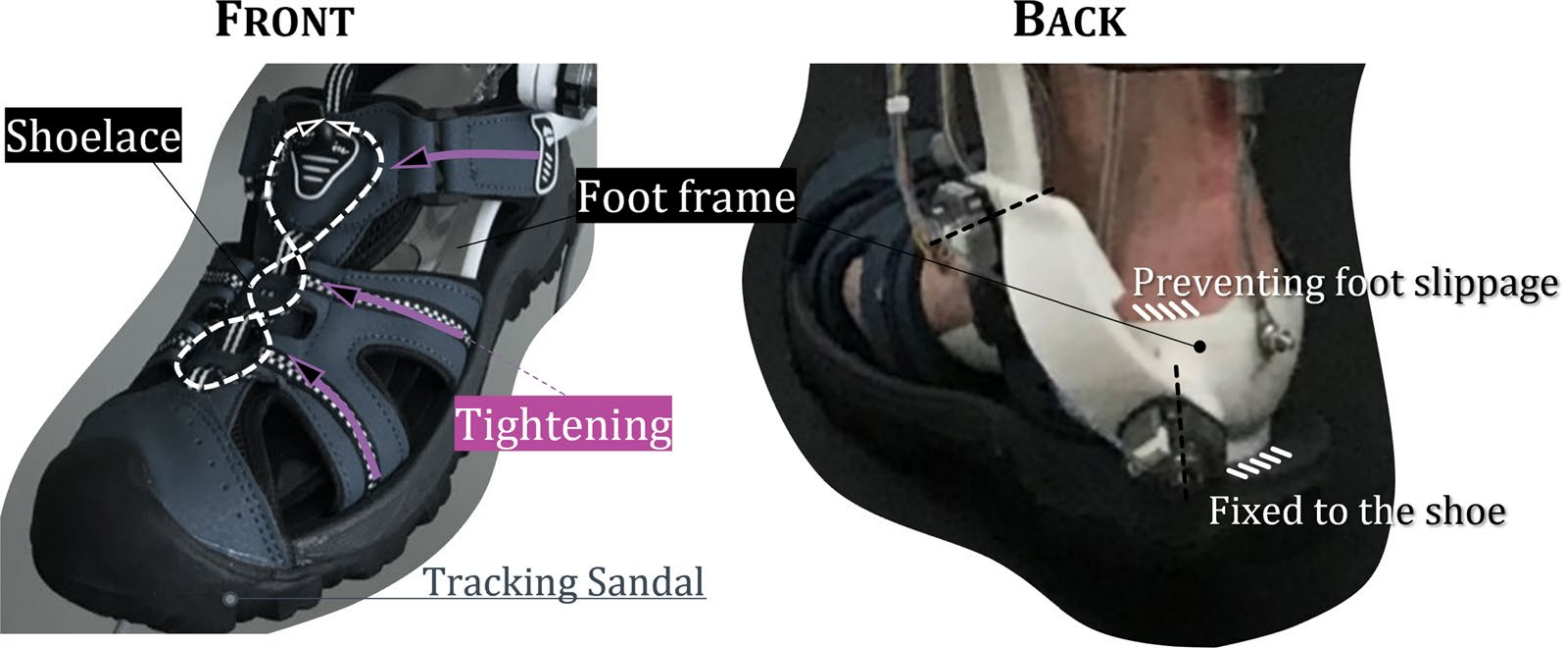
Description of the part for interface between foot and PAFO

We used previously developed 2 *dof* PAFO for clinical experiments [21]. The developed 2 *dof* PAFO has talocrural and subtalar joints as rotational axes, and they were calculated based on anatomical data (Fig. 1a). This PAFO interfaces with the wearers at the foot, calf and thigh. For calf and thigh, straps are used for interface. In the case of the foot, tracking sandal was used for tightening the whole foot using shoelace (Fig. 2). Two PAMs were used as actuators, which simultaneously assist plantar flexion and eversion. Due to the nature of PAM, only contraction force can be provided, and verification of whether they have sufficient force and workspace within their contraction capacity is detailed in previous study. Its frames were fabricated with 3D printers, and the total weight is 1.44 kg excluding knee orthosis. This value is smaller than the average weight of assistive PAFO of 1.69 kg, and its mass breakdown is also detailed in the previous study.

#### Foot slippage

Since we have a plan to design a controller using CoP location and use it in our experiments, the reliability of its measurement is very important. In order to measure CoP accurately, it is important to fully realize the interface between the foot and the PAFO. In the case of our developed PAFO, since the foot frame is fabricated based on the 3D scanned foot model, it is designed to cover the heel as shown in Fig. 2. And the foot frame is fixed to the shoe so that slippage of the foot can be minimized. And we made PAFO with tracking sandal which is in a form that wraps the foot and the foot frame. So, when the shoelace is pulled, the entire interface is tightened as shown in Fig. 2, increasing the rigidity of the bond between the foot—foot frame—tracking sandal, and almost eliminating the occurrence of the foot slippage.

### Controllers for assistance

**Fig. 3:**
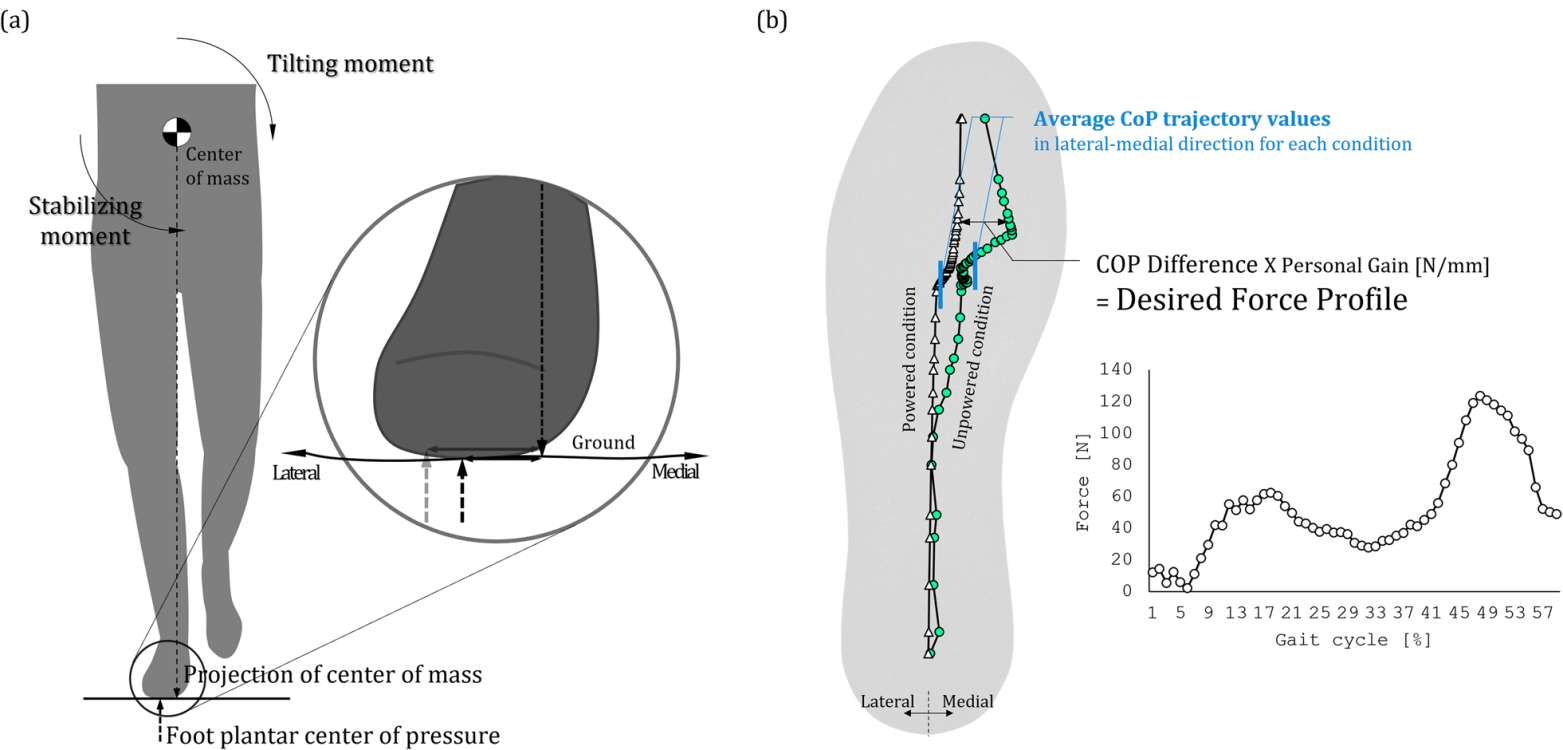
**a** Explanation of the ankle strategy for changing the stabilizing and tilting moments, **b** Force profile for the CoP difference controller and representation of the average CoP trajectory

Since two actuators are included, two controllers are required for each PAM during the human experiment. In a previous study, we found that the power provided by PAM1 causes the in-shoe CoP to move in the lateral direction, which leads to an increase in the length of the postural path [22]. And in the case of the 1 *dof* PAFO, eversion was impossible to implement, and this phenomenon was prominent. When the CoP moves laterally, the distance between the plantar CoP and the projection of the CoM increases, and then the tilting moment increases in proportion to this change, as revealed in previous studies (Fig. 3a). This leads to an increase in postural sway. Therefore, we tried to develop a controller for PAM2 that could reduce the tilting moment by generating a stabilizing moment proportional to the distance the CoP moves in the lateral direction.

First, subjects wearing the 2 *dof* PAFO walked on the treadmill while PAM2 was in the unpowered condition, and a phase-based controller (PBC) was applied only to PAM1. The PBC operates by determining the phase of the gait cycle and then setting the positive power onset for a certain time, and it is used for many PAFOs [8]. After identifying heel strike using a force-sensitive resistor (FSR) sensor built into the PAFO, we opened the solenoid valve at a certain time so that the onset of positive power was located between 40 and 50% of the gait cycle. This value has been proven to be the optimal timing for activating a PBC in previous studies [9, 32, 33]. Experiments were carried out for 4 cases in which the solenoid had duty ratios of 30%, 60%, and 90% for PAM1, and an experiment in which both PAM1 and PAM2 were unpowered was also conducted. During experiments, an insole pressure sensor (MP2512PLUS; Kitronyx Inc, Seoul, Korea) was installed under the tracking sandal which is shown in Fig. 1c, and using this, we were able to track the CoP trajectory. Experimental data showed that the trajectory of the CoP was located more laterally when PAM1 was actuated than during the unpowered conditions, and the force profile, which was obtained by calculating the differences in trajectories between the powered and unpowered experiments, is shown in Fig. 3b.

**Fig. 4:**
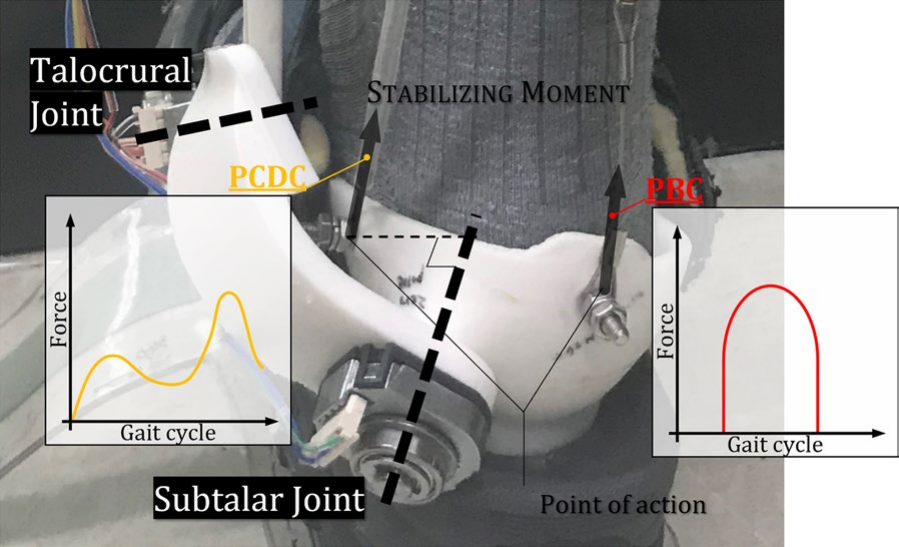
Each controller is used for two PAMs

The obtained profile indicates the magnitude of the increasing tilting moment as well as the difference in the CoP trajectory between the powered and unpowered conditions [28]. We hoped to actuate PAM2, which strengthens the rotation of the subtalar joint in the direction of eversion, in proportion to this profile to assist the stabilizing moment in preserving whole body stability according to the ankle strategy. We named this the proportional CoP difference controller (PCDC). We designed a total of three controllers, PCDC-A, PCDC-B, and PCDC-C, corresponding to the three duty ratios (30%, 60%, 90%) provided to PAM1 in the experiment. Of course, we cannot guarantee that these controllers are optimal, but we expected that they would be sufficient to analyze the role of a 2 *dof* PAFO with a subtalar joint in gait stability. While a PCDC was used for PAM2, a PBC was used for PAM1, as shown in Fig. 4, and the duty ratio was set to the same value as that of the PCDC.

### Measurements

#### Postural sway

First, a stabilogram was used to measure postural sway [34]. A stabilogram is a method of assessing a subject’s postural sway by plotting the acceleration or angular acceleration of the trunk as measured by an accelerometer or gyroscope in 2D or 3D space [35]. We attached an inertial measurement unit (IMU) to the wearer’s trunk, as shown in Fig. 1c, and measured the roll and pitch angles during the experiments [36]. We plotted the measured data in 2D space, and the Gaussian method was applied to the plotted measured data. Then, we obtained the standard deviation (SD) values for the scatter data from the stabilogram, and their root mean square (RMS) values were used as indices of postural sway. Postural pathways were calculated during the whole gait cycle and the stance phase (0–50% of the gait cycle).

#### Kinematic variability

Kinematic variability is an index used to evaluate gait stability and is quantified as the mean SD of data for a cyclical motion [26, 37]. It involves calculating the variability between cycles of the data trajectories (i.e., SD) and the average values during the cycle. We used inertial measurement unit (MPU6050, TDK InvenSense) for measuring 3-axis acceleration of the trunk as shown in Fig. 1c and its data was acquired through STM32F4DISCOVERY with 1000 Hz. We normalized the 3-axis acceleration values to the percentage of the gait cycle and calculated the mean SD for each. After that, it was converted to a ratio of the values from the unpowered condition, and the RMS values of the individual axes were calculated and used as a value representing one case. Kinematic variabilities were also calculated during the whole gait cycle and the stance phase (0–50% of the gait cycle) so that we could analyze the effects of the 2 *dof* PAFO during one-leg stance when the ankle strategy was used.

#### Local dynamic stability

LDS was used as an indicator of stability in the study of Rosenstein et al. and in gait analysis in the study of Dingwell [25, 38], and the short-term largest Lyapunov exponent (LLE) of the gait cycle has been used to predict the risk of falling and the patient’s condition. Therefore, we also included this index and used the 3-axis acceleration data from the IMU to calculate the LLE. IMU data were normalized by heel contact signals, and a divergence curve was calculated with them. The 0–0.5 stride section was chosen for calculating the slope of the mean divergence curves using a linear square fit, and these values were used as the short-term LLEs, which represent the gait stability of the subjects.

#### Electromyography

The fundamental function of a PAFO is to reduce MR or EMG activity by assisting the wearer’s movements. Therefore, we used surface EMG sensors (AM530, LAXTHA Inc.) attached to the soleus muscles of the subjects to properly evaluate the assistance and its data was acquired through STM32F4DISCOVERY with 1000 Hz. The acquired EMG data were converted into integrated EMG (iEMG) data to represent one gait cycle. The average of the iEMG values for all gait cycles was calculated and used as a representative value of the experimental results of each case for each controller.

#### Kinematic variables based on PAFO sensors

The developed PAFO includes various sensors that can monitor the wearer’s kinematics, as shown in Choi et al. First, the rotation angle of the talocrural and subtalar joints can be measured through the absolute encoder. By substituting these values into the rotation matrix, the contraction length of the artificial muscles can be estimated, and the power provided to the wearer can be calculated by multiplying them by the value of the loadcell installed on the wire connecting the PAMs and the frames. Similar to the iEMG data, the PAFO power was also normalized for each cycle, and the positive power applied to the wearer was integrated and converted into an average positive power for an entire gait cycle. The abovementioned in-shoe pressure sensor for the PCDC controllers was also used to determine the CoP trajectory. In addition, an FSR sensor was installed in the lower part of the sole, and all data from it were normalized to one gait cycle and used to calculate the timing of the controllers.

### Experimental setup

**Fig. 5:**
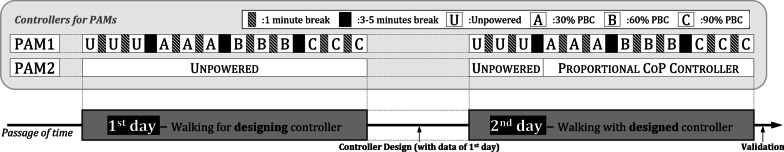
Experimental protocol

A total of 7 subjects (174 ± 7 cm, 71 ± 5 kg, 30 ± 4 years) participated in the experiment. The number of participants was determined by referring to the average number of participants in previous PAFO experiments [9, 33, 39–42] so that similar statistical power could be obtained. All experimental procedures were carried out with the approval of the Institutional Review Board of Yonsei University (7001988-202003-HR-833-03), and the participants were given verbal and written explanations about the experimental process and contents. All participants were recruited randomly by recruitment notice, so it was true experimental study. And they are healthy and had never experienced gait abnormalities or related diseases. In fact, they participated our previous experiments [22], so they are already familiar with developed PAFO. Experiments were conducted on two different days and the protocol is described in Fig. 5. On the first date, participants walked on the treadmill at a speed of 1.4 m/s with PBC only for PAM1 (unpowered and 30%, 60%, 90% of duty ratio) for designing the PCDC. Three sets were performed for each controller, and each set consisted of walking for approximately maximum 5 min (50 steps, 3–5 min). In addition, to minimize participant fatigue, a 1-min rest period between sets and a 3-min rest period between controllers were provided. Experimental data was used for designing the controllers in the middle of the 2 days for experiments, and PCDCs for each participant were designed for 2nd day. And on the 2nd day, same participants conducted the walking experiments on the treadmill with same procedures and the newly developed user-specific controllers (PBC for PAM1 and PCDCs for PAM2) as shown in Fig. 4 for the validation.

### Statistical analysis

All data were converted to a percentage of the value of the unpowered condition for group analysis. The average value measured in the three trials of the unpowered condition was used to calculate the ratio of the data of the unpowered condition, PCDC-A, PCDC-B, and PCDC-C. In the case of the eversion angle, the difference between each angle and the mean eversion angle in the unpowered condition was used for comparison, and all cases again had one mean and SD. For comparisons between cases, we used Wilcoxon signed-rank test of Python’s SciPy package because of low sample size. And since multiple comparisons were conducted for a total of 4 groups including the unpowered condition, the difference between the groups was judged by corrected significant level of 0.008 through Bonferroni method. Pearson’s correlation analysis was used to analyze the correlation between the results, and partial correlation analysis was performed to determine the effects of PAMs on the measurement values by leave-one-out analysis.

## Results (Fig. 6)

**Fig. 6:**
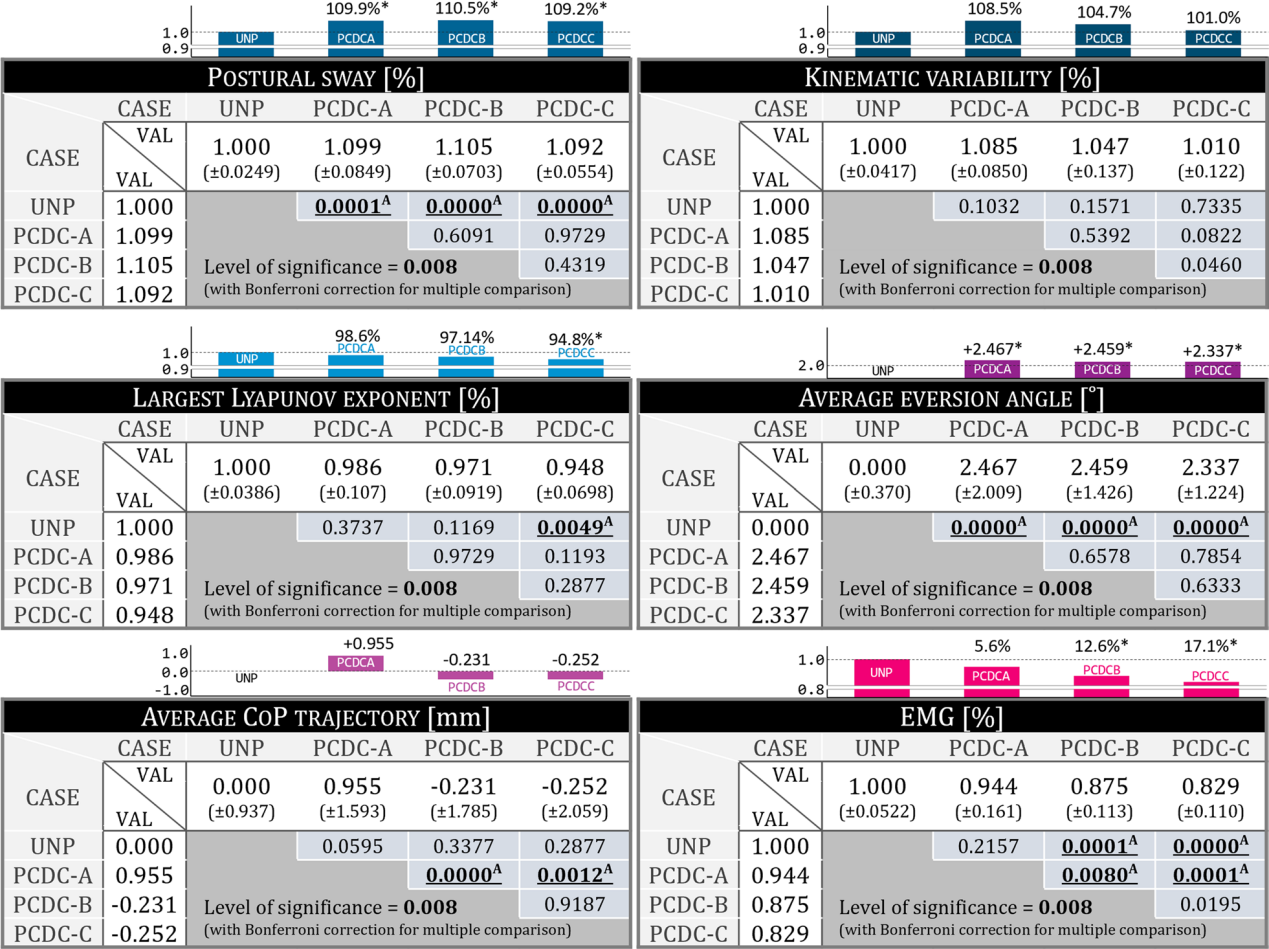
Summary of all measured data with means and standard deviations. *Indicates a significant difference compared to unpowered conditions. ^A^ means that there is a significant difference between the two. UNP indicates the unpowered condition

### Postural sway

The stabilogram results are expressed as a ratio, where the average value of the results of the unpowered condition is 1. The ratios for the PCDC-A, PCDC-B, and PCDC-C conditions were 1.099, 1.105, and 1.092, respectively, suggesting that the postural sway in the powered condition increased more with respect to the unpowered condition (p < 0.008). No significant difference was found among the powered conditions.

### Kinematic variability

For the PCDC-A, PCDC-B, and PCDC-C conditions, the values were 1.085, 1.047, and 1.010, respectively, but no statistically significant difference compared to unpowered condition was found.

### Local dynamic stability

The short-term LLE ratios for the PCDC-A, PCDC-B, and PCDC-C conditions were 0.986, 0.971, and 0.948, respectively. The results of the Wilcoxon signed-rank tests indicate that wearers are more stable during the PCDC-C condition than during the unpowered condition (p < 0.008).

#### Average eversion angle

Average eversion angle was calculated from the average angle change during the gait cycle for each stride. From the experimental results indicated stronger eversion during the powered conditions than during the unpowered conditions. When the average value of the eversion angle profiles in the unpowered condition was 0, the results for PCDC-A, PCDC-B, and PCDC-C were larger by 2.467°, 2.460°, and 2.337°, respectively, and these were statistically significant differences compared to unpowered condition (p < 0.008).

#### Average center of pressure trajectory

The average CoP trajectory value, which represents the average value of the CoP trajectory in the lateral-medial direction during one gait cycle, was 0.955, − 0.231, and − 0.252 for PCDC-A, PCDC-B, and PCDC-C, respectively, when that of the unpowered condition was 0. The Wilcoxon signed-rank tests confirmed that three powered conditions had no significant differences compared to unpowered conditions.

#### Electromyography

PCDC-A, PCDC-B, and PCDC-C were associated with EMG reductions of 5.6, 12.6 and 16.1%, respectively, compared to the unpowered condition, and these reductions were all statistically significant (p < 0.008). Additionally, it was found that PCDC-B and PCDC-C were associated with greater reductions in EMG activity than PCDC-A.

### Correlation coefficient

**Fig. 7:**
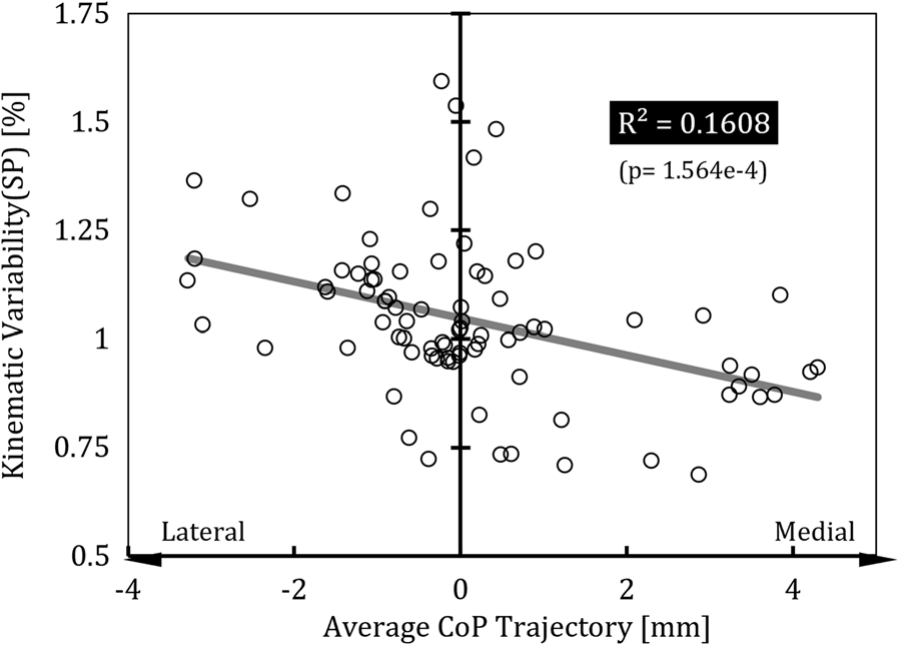
Correlation between the average CoP trajectory and kinematic variability during the stance phase based on Pearson’s correlation analysis. Kinematic variability is calculated as the ratio with respect to the results of the unpowered condition, and the values of the average CoP trajectory for the powered conditions are different from the results for the unpowered condition. SP indicates the stance phase

Several moderate correlations (${R}^{2}>0.1$) were found between the measurement results and the average positive power of PAM1, PAM2 or their sum, as shown in Table 1. PAM1 had a positive correlation with postural sway, which indicates that an increase in the power of the PAM results in an increase in postural sway. And no significant relationship was found for PAM1 with the rest of the measurement. As we intended, the power of PAM2 enhanced the eversion angle, and the correlation was positive and rest of the measure values showed no significant relationship. The average CoP trajectory was negatively related to the average eversion angle (R = − 0.329 and p value = 2.277e−3), as shown in Table 2. The average CoP trajectory also had some correlation with stability indices, namely, kinematic variability (Fig. 7), during the stance phase. The results of the partial correlation analysis in Table 3 show that PAM1 is related to an increase in postural sway (p < 0.05) and PAM2 is associated with enhanced eversion and CoP shifts in the medial direction (p < 0.05). EMG activity had a negative correlation with the sum of the powers of the PAMs (p < 0.05), which indicates that the power of the PAMs generated proper propulsion for the subjects.

**Table 1:** Results of Pearson’s correlation analysis for the PAMs

	PAM1		PAM2		PAM1 + PAM2	
	R	p value	R	p value	R	p value
Postural sway	0.369^A^	5.511e−4	0.276	1.096e−2	0.451^A^	1.641e−5
Kinematic variability	0.177	1.065e−1	0.175	1.121e−1	0.243	2.598e−2
LLE	− 0.083	4.533e−1	0.078	4.787e−1	− 0.014	8.986e−1
Average eversion angle	0.149	1.748e−1	0.590^A^	3.433e−9	0.480^A^	3.797e−6
CoP	− 0.149	1.752e−1	− 0.301	5.375e−3	− 0.300	5.505e−3
EMG	− 0.128	2.464e−1	− 0.363^A^	6.990e−4	− 0.322^A^	2.791e−3

A means that $${R}^{2}>0.1$$

**Table 2:** Results of Pearson’s correlation analysis for the CoP

	Postural sway	Kinematic variability	Postural sway (SP)	Kinematic variability (SP)	LLE
CoP					
R	− 0.109	− 0.279	0.318^A^	− 0.401^A^	− 0.237
p	3.252e−1	1.029e−2	3.216e−3	1.564e−4	2.988e−2

A means that $${R}^{2}>0.1$$

**Table 3:** Results of the partial correlation analysis for the PAMs

	Postural sway	Kinematic variability	Postural sway (SP)	Kinematic variability (SP)	LLE	Average eversion angle	CoP	EMG
PAM1								
R	0.372^A^	0.173	0.323^A^	0.179	− 0.0866	0.155	− 0.143	− 0.121
p	4.891e−4	1.159e−1	2.744e−3	1.026e−1	3.336e−1	1.605e−1	1.930e−1	1.930e−1
PAM2								
R	0.281	0.170	0.0163	0.185	0.0822	0.591^A^	− 0.298	− 0.361^A^
p	9.676e−3	1.221e−1	8.827e−1	9.157e−2	4.573e−1	3.215e−9	5.820e−3	7.548e−4

A means that $${R}^{2}>0.1$$

## Discussion

We determined the effects of a 2 *dof* PAFO with talocrural and subtalar joints as rotational axes, with an ergonomic design and configuration similar to that of human ankle joints, on wearer gait stability with respect to the ankle strategy using clinical experiments. For this, a PBC, which was used basically as a controller, was used for PAM1, which is mainly responsible for propulsion, and the PCDC was designed so that PAM2 could strengthen the eversion and stabilizing moment of the ankle strategy. Gait experiments were conducted on subjects wearing the 2 *dof* PAFO with these controllers. To briefly summarize the experimental results, even if the size of the power provided by the 2 *dof* PAFO increased, only postural sway increased, but no significant increase in kinematic variability was observed. In the case of LDS, the smallest value was observed with the PCDC-C, which indicates that the gait stability was better despite the magnitude of the provided power and the noticeable decrease in EMG activity.

First, looking at the results for the average eversion angle (Fig. 6), it can be seen that the eversion was significantly strengthened in the powered condition compared to the unpowered condition. The average eversion angle had a positive correlation (0.480) with the sum of the power of PAM1 and PAM2. It also had a partial correlation (0.591) with PAM2, which indicates that PAM2 has larger effects on eversion than PAM1 consistent with its design purpose. It is also possible that this was not only accomplished by the power provided to the subjects but by wearers being able to implement the ankle strategy more freely during the one-leg stance period when the subtalar joint was added to the PAFO. Thus, when the 2 *dof* PAFO provides power, the wearer implements a different ankle strategy than during the unpowered condition, and the movement strategy also varies. It is unknown which had more influence, but eversion was strengthened when power was provided to the subjects.

Looking at the results of the average CoP trajectory (Fig. 6), there was no difference between the unpowered and PCDC-B or PCDC-C conditions, which provided power. In a previous study, we found that in the case of gait with a 1 *dof* PAFO, the average CoP trajectory is located in more laterally as the power provided by the PAM increases, and postural sway increases in proportion to the increase in the tilting moment [22]. Comparing these results, it can be seen that the ankle strategy was implemented or strengthened due to the presence of the subtalar joint or the assistance of the PAMs, and thus, the CoP could be maintained in the same position as in the unpowered condition. A negative correlation was found between the average positive powers of the PAM2 and the average CoP trajectory (Pearson = − 0.301, partial = − 0.298), supporting this claim.

The reason that the correlations between the magnitude of power provided in the powered condition and the average eversion angle or average CoP trajectory were not strong may be due to a combined effect of the provided power on them (Table 1). When one foot is on the ground during one-leg stance and subjects are assisted by PAMs, the stabilizing moment is enhanced if its eversion angle is maintained at a certain level by stiffening the ankle joint with co-contraction. This moment acts in the opposite direction to the tilting moment caused by the misalignment between the projection of the CoM and the plantar CoP, and it affects the position of the CoM, reducing the size of the misalignment between the two. Therefore, it can be inferred that the correlation was not clearly observed because the power of the PAMs was divided and used to reduce the tilting moment by enhancing the eversion or strengthening the stabilizing moment. Additionally, some of the power was used to reduce EMG activity, with similar efficiency to other PAFOs [10, 43, 44].

Postural sway was significantly increased in the powered condition compared to the unpowered condition as shown in Fig. 6. Although there was a difference between the powered and unpowered conditions, this is a meaningful result when compared to those for a 1 *dof* PAFO as shown in previous study [22]. In the case of the 1 *dof* PAFO, postural sway increased in proportion to the power provided, but there was no trend or correlation found with the 2 *dof* PAFO. The possibility of executing an ankle strategy can be considered to have contributed to this. Otherwise, no significant difference was found in the measured kinematic variability among any of the cases. These results are different from those of postural sway, which increased with the power of the PAM provided in the powered condition, and it can be considered that even if the postural sway increased, when the ankle strategy was properly implemented, it did not lead to an increase in kinematic variability. Kim and Collins determined the characteristics of propulsion in amputees using an ankle–foot prosthesis using frontal plane dynamics and found that the use of controllers reduced step width variability [3]. Although they did not measure trunk variability, step width is related to stability, and their results are consistent with ours in that the variation in frontal plane rotation between cycles improved when ankle movement was assisted in the frontal plane. These results are also aligned with those of the study of van Leeuwen, which verified that the shifting of the CoP in the lateral-medial direction achieved with the ankle strategy is important for compensating for the error of foot placement during the stance phase in achieving stable walking. Therefore, it can be argued that even if the wearer receives power from the PAFO and then the outward fluctuation increases due to the increase of the postural sway, it does not lead to deterioration of gait stability if characteristics of assistance supports movement in the frontal plane and enables an appropriate ankle strategy.

The correlation between the average CoP trajectory and kinematic variability in the stance phase was negative, as shown in Fig. 7, which means that when the in-shoe CoP moves in the medial direction in the local coordinates of the sole, gait stability improves. This can be explained by the fact that the tilting moment decreasing because the distance between the plantar CoP and the projection of the CoM decreased [17]. Therefore, the subtalar joint allows the wearer to more freely implement the ankle strategy, and when PAM2 enhances eversion or the stabilizing moment, the in-shoe CoP stays in a more medial location so that the tilting moment does not increase and stable gait can be established. Unlike kinematic variability, postural sway had a positive correlation with the average CoP trajectory. The explanation for this is unclear, but we know that the CoP is not directly related to body sway but to the tilting moment [17], so we can guess that the positive correlation between postural sway and the power provided by PAM1 results from a change in movement strategies when walking with assistance, although the change does not affect gait stability if an ankle strategy can be implemented. In the case of LLEs, another index of stability, the average value tended to decrease as the amount of power provided to the PAM increased, and the result for the PCDC-C condition, which provided the greatest power, showed that very stable walking was achieved compared to the unpowered condition. Although the correlations between the LLE and the average positive powers of PAM1 and PAM2 were not clearly shown, the most stable gait was achieved under the PCDC-C condition, which involved the greatest power. This shows that the 2 *dof* PAFO not only reduced EMG activity but also appropriately supported implementation of the ankle strategy, resulting in a stable gait, which indicates that the assistance has been provided allowed for the maintenance of gait stability despite the increase in postural sway.

Hof identified three mechanisms by which balance can be maintained by calculating the equation of motion for humans during one-leg stance [18]. They are (1) moving the plantar CoP, (2) counter-rotating, and (3) applying an external force. Based on the experimental results, we found that the 2 *dof* PAFO assisted the ankle strategy via all three mechanisms. First, unlike the 1 *dof* PAFO, it enabled eversion through the subtalar joint to move the plantar CoP. Through the actuation of PAM 1 and 2 (applying external forces), the compensation of the stabilizing moment for the tilting moment caused by the difference between the projection of the CoM and the plantar CoP was strengthened, making it possible to properly implement counterrotation. In view of this analysis, it can be argued that the subtalar joint is an essential element in PAFOs because it enables subjects using PAFOs to implement an ankle strategy and enables stable walking adjusting movements in the frontal plane.

Our study has several limitations. First, we did not measure gait stability when walking with a 1 *dof* PAFO, where an ankle strategy cannot be implemented. However, a previous study showed that the average CoP trajectory is located more laterally in proportion to the power provided and that there is a tendency towards increased postural sway because the ankle strategy cannot be implemented due to its physical limitations. From this, it can be predicted that the gait will not be stable because the increase in the tilting moment caused by the CoP location is obvious and an ankle strategy cannot be implemented, so the adverse effect of postural sway on gait stability cannot be alleviated. If gait stability did not deteriorate at all by taking a wide step or by changing the other joint movements, qualitative rehabilitation would not be utilized because wearers cannot recover sufficient proprioception for implementing the ankle strategy.

The second limitation is that walking without a PAFO was not included in the experiment. We did not include the condition of not wearing a PAFO at all because we focused on showing whether the power provided by the PAFO properly implements eversion and the ankle strategy, which cannot be accomplished with a 1 *dof* PAFO, and tried to show its importance. Therefore, there are no data to determine whether the gait is better than when the 2 *dof* PAFO is not worn. Although the weight of the PAFO is 1.44 kg which is lower than average value of the previously developed PAFOs, its weight could affect walking of the participants. And they had enough time to rest between the trials to prevent fatigue but it is possible that the wearing of PAFO had some effect on them. However, we found that implementation of the ankle strategy affects the gait stability of subjects when PAFOs are powered, and this is a meaningful result for PAFO fabrication and experimentation.

Last limitation is that we did not prove experimentally that foot slippage did not occur. According to previous studies, foot slippage was observed in 2 *dof* AFO [45, 46], and since we used CoP as important index, when this phenomenon occurs, the experiment is negatively affected. However, to prevent this phenomenon, we used tracking sandal as an interface between the foot and PAFO, and since the foot frame is in the shoe, so when the shoelace is tight, the foot can be tightly attached to the frame to minimize slippage. Nevertheless, there is a possibility that foot slippage may occur, so we intend to specify this as a limitation.

As we proceeded to analyze the results of this study, we had some suggestions for further studies. First, since the PCDC is pre-defined and not an optimal controller, if we find some controllers or force profiles that can better provide a stabilizing moment, we can maximize the effect of 2 *dof* PAFO. It can be an adaptive PCDC which changes in realtime based on the measured CoP position, or a human-in-the-loop algorithm that considers the gait cycle as a iteration and optimizes the indices of the force profile may be used with objective functions of minimizing the kinematic variability or moving average of LLE. And, if the tilting moment is measured in more detail using a 3D motion capture camera and the direction of the provided power in global coordinates can be calculated, the principle of the 2 *dof* PAFO can be understood more deeply by comparing those results. Lastly, if we design a device or method that can measure foot slippage so that minimize the inaccuracy or error of the CoP measurement, the effectiveness of the designed controller can also be improved.

## Conclusions

We conducted a gait experiment using a 2 *dof* PAFO that has both talocrural and subtalar joints as rotation axes to analyze the effect of ankle strategy implementation on the gait stability of wearers. And we also designed and used the PCDC which provides frontal plane powers proportional to the magnitude of the tilting moment predicted based on the CoP position in the medial–lateral direction. It was shown that subjects implemented an ankle strategy by reinforcing rotation about the subtalar joint in the powered condition and that the CoP trajectory could be maintained in the same position as in the unpowered condition. Additionally, as revealed in previous studies [22], postural sway did not increase in proportion to the provided power also at this time and no significant difference was found in kinematic variability which represents the gait stability between the unpowered and powered conditions though this study. The LDS results also showed that more stable walking can be achieved under powered conditions where EMG decreased most. These facts suggest that gait stability can be preserved even with increase in postural sway if an appropriate ankle strategy is implemented. And it proves that the ankle strategy is an important component of gait stability even when walking with PAFO assistance, and it can be argued that the subtalar joint is an essential element for providing stable assistance with PAFOs.

## Data Availability

Data and scripts will be made available by the corresponding author upon reasonable request.

## Abbreviations

PAFO: Powered ankle–foot orthosis
*dof*: Degree of freedom
CoM: Center of mass
CoP: Center of pressure
PAM: Pneumatic artificial muscle
PBC: Phase based controller
PCDC: Proportional CoP controller
IMU: Inertial measurement unit
SD: Standard deviation
RMS: Root mean square
LLE: Largest Lyapunov exponent
iEMG: Integrated electromyography
